# HPLC-HRMS Quantification of the Ichthyotoxin Karmitoxin from *Karlodinium armiger*

**DOI:** 10.3390/md15090278

**Published:** 2017-08-31

**Authors:** Aaron John Christian Andersen, Lívia Soman de Medeiros, Sofie Bjørnholt Binzer, Silas Anselm Rasmussen, Per Juel Hansen, Kristian Fog Nielsen, Kevin Jørgensen, Thomas Ostenfeld Larsen

**Affiliations:** 1 National Food Institute, Technical University of Denmark, Kemitorvet Building 202, 2800 Lyngby, Denmark; ajca@food.dtu.dk (A.J.C.A.); kejo@food.dtu.dk (K.J.); 2 Department of Biotechnology and Biomedicine, Technical University of Denmark, Søtofts Plads, Building 221, 2800 Lyngby, Denmark; silan@bio.dtu.dk (S.A.R.); kfn@bio.dtu.dk (K.F.N.); 3 Departamento de Química, Universidade Federal de São Paulo (UNIFESP), Rua São Nicolau, 210, CEP 09913-030 Diadema-SP, Brazil; liviasoman@gmail.com; 4 Marine Biological Section, Department of Biology, University of Copenhagen, Strandpromenaden 5, 3000 Helsingør, Denmark; sofie.binzer@bio.ku.dk (S.B.B.); pjhansen@bio.ku.dk (P.J.H.)

**Keywords:** harmful algal bloom, ichthyotoxic, karlotoxin, amphidinol, polyether, polyketide, quantify, quantitation

## Abstract

Being able to quantify ichthyotoxic metabolites from microalgae allows for the determination of ecologically-relevant concentrations that can be simulated in laboratory experiments, as well as to investigate bioaccumulation and degradation. Here, the ichthyotoxin karmitoxin, produced by *Karlodinium armiger*, was quantified in laboratory-grown cultures using high-performance liquid chromatography (HPLC) coupled to electrospray ionisation high-resolution time-of-flight mass spectrometry (HRMS). Prior to the quantification of karmitoxin, a standard of karmitoxin was purified from *K. armiger* cultures (80 L). The standard was quantified by fluorescent derivatisation using Waters AccQ-Fluor reagent and derivatised fumonisin B_1_ and fumonisin B_2_ as standards, as each contain a primary amine. Various sample preparation methods for whole culture samples were assessed, including six different solid phase extraction substrates. During analysis of culture samples, MS source conditions were monitored with chloramphenicol and valinomycin as external standards over prolonged injection sequences (>12 h) and karmitoxin concentrations were determined using the response factor of a closely eluting iturin A2 internal standard. Using this method the limit of quantification was 0.11 μg·mL^−1^, and the limit of detection was found to be 0.03 μg·mL^−1^. Matrix effects were determined with the use of *K. armiger* cultures grown with ^13^C-labelled bicarbonate as the primary carbon source.

## 1. Introduction

Harmful algal blooms (HAB) are a global phenomenon with an apparent increasing occurrence [1,2,3]. Their adverse effects to coastal communities, public health, tourism, and fisheries can be devastating and have large economic implications [4]. In addition to causing adverse effects on people exposed to toxins accumulated in shellfish [5,6], HAB are also responsible for wide spread mortalities in a range of marine and marine-associated species [7,8,9,10], these events can indirectly affect human populations via economic loss in farmed fish, as well as through the death of fish and shellfish in wild fisheries [4]. 

Although microalgae are known producers of toxins that can affect humans, such as saxitoxin responsible for paralytic shellfish poisoning [11,12,13], okadaic acid responsible for diarrhetic shellfish poisoning [14,15], and domoic acid responsible amnesic shellfish poisoning [16], the ichthyotoxic metabolites and mechanisms responsible for fish kills during HAB events are not so well understood [17]. Recently, however, the mode of action for karlotoxin 8, one of many structurally-related metabolites produced by dinoflagellates, has been investigated by NMR and suggests that these toxins bind to cell membranes in a hairpin-type arrangement, causing lysis [18]. It is suspected that the variation in the intramolecular polarity of these metabolites, having both polar and apolar regions, assists in their toxicity [18]. It may also explain the characteristic of these medium polarity metabolites being retained on polytetrafluoroethylene (PTFE) filters as well as on glass fibre filters [19].

For common algal toxins which affect humans there are numerous quantitative and semi-quantitative methods, however, for ichthyotoxic metabolites there are few [19,20], with the exception of ichthyotoxins, which also effect humans. This is certainly the case for the brevetoxin and ciguatoxin class of neurotoxins, which are routinely monitored with enzyme-linked immunosorbent assays and high-performance liquid chromatography (HPLC) coupled to tandem mass spectrometry (MS/MS) [21,22,23,24]. For the linear superchain polyether class of microalgae metabolites, such as karlotoxin 2, only low-resolution MS methods have been reported for laboratory-grown cultures [19]. There are many challenges in working with these compounds, in addition to the lack of commercially-available standards, such as difficulties in strain cultivation and harvesting on large scales, low biological production of the ichthyotoxins, compound degradation, and biomass/media split of ichthyotoxins.

MS/MS analysis on triple quadrupole mass analysers (QqQ) is the dominant trace quantification technique due to the high sensitivity and selectivity in selected reaction monitoring (SRM, also known as multiple reaction monitoring, MRM). However it has been shown that accurate mass quadrupole time-of-flight MS (QTOF-MS) instruments can provide selectivity comparable to precursor-product ions and ratios from SRM experiments [25,26]. In addition to this, although the sensitivity of QTOF-MS instruments may not match that of QqQ instruments, they can come close and, additionally, they allow both targeted identification as well as screening for unknowns, which can be beneficial when analysing environmental samples, detecting metabolites of biotransformation, as well as recursive data-analysis [27,28,29].

The dinoflagellate *Karlodinium armiger* produces karmitoxin [30], an ichthyotoxic metabolite structurally related to the karlotoxins, amphidinols, and others (Table S1) [31,32,33,34,35,36,37,38,39,40,41,42,43,44,45,46,47,48,49,50,51,52,53,54,55]. Presented here is a method for the quantitative analysis of karmitoxin in laboratory-grown cultures of *K. armiger* using iturin A2 as an internal standard by HPLC-QTOF-MS. The method developed assessed nine different sample preparation procedures, including six solid phase extraction (SPE) substrates, and source conditions were monitored with external reference standards, valinomycin and chloramphenicol. To avoid compound loss in the sample preparation procedures, whole culture subsamples were extracted, eliminating the possibility of toxin transfer to the aqueous phase by cell lysis during centrifugation, and also capturing any allelopathic proportion of karmitoxin. Recovery and matrix effects were also assessed by standard addition to ^13^C-labelled cultures. A quantitative standard of karmitoxin was purified from laboratory-grown cultures of *K. armiger* and quantified by fluorescent derivatisation in reference to a calibration curve of derivatised fumonisin B_1_ and fumonisin B_2_. Other studies have shown that high growth rates of *K. armiger* can be achieved in mixotrophic cultures, however, it is not known how toxin production changes in these mixotrophic environments [56]. The relationship of karmitoxin concentration and the growth phase of *K. armiger* was investigated, as well as a comparison of karmitoxin concentration in cultures grown under phototropic conditions with cultures grown under mixotrophic conditions.

## 2. Results and Discussion

### 2.1. Method Development

**Figure 1:**
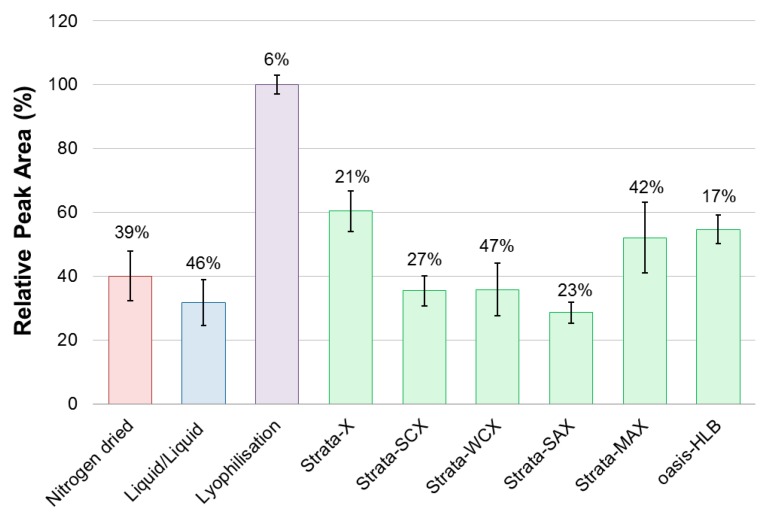
Performance of sample preparation procedures. Integrated peak areas of karmitoxin (peak area of the monoisotopic isotopomer from the most abundant adduct, [M + H]^+^), from HPLC-QTOF-MS analysis of different sample preparation methods and relative standard deviations of the five replicates (three replicates for solid phase extraction methods, indicated collectively with green bars) from each sample preparation procedures.

Of the nine different sample preparation methods, it was found that lyophilisation resulted in the greatest integrated peak area relative to extraction volume and also had the lowest standard deviation of all sample preparation methods (Figure 1).

Nitrogen drying and liquid/liquid sample preparation methods both had poor repeatability and relatively low relative recovery. In terms of sample preparation performance, Strata-X SPE had the greatest relative recovery of all SPE methods and one of the lowest relative standard deviation (RSD). Among all sample preparation methods lyophilisation had the greatest relative recovery, as well as the lowest RSD, however, due to concerns about matrix effects, an SPE method was also utilized in further analysis. The two methods with the greatest relative recoveries, lyophilisation and Strata-X SPE, were chosen as the preparation methods for laboratory experiments. 

Monitoring source conditions, in terms of adduct pattern stability of external standards over time, during analysis of samples prepared using lyophilisation and Strata-X, it was found that there was significantly greater stability in the adduct formation of external standards during the analysis of samples prepared using Strata-X. This stability was likely due to the better removal of salts.

**Figure 2:**
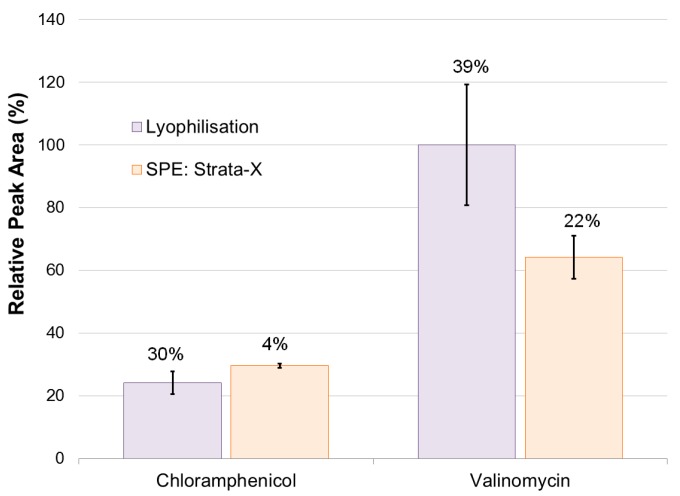
Combined integrated peak areas of monitored adducts for each of the external standards: chloramphenicol ([M + H]^+^, [M + Na]^+^, [3M + Fe(II)]^2+^, [2M − 2H + Fe(III)]^+^) and valinomycin ([M + H]^+^, [M + NH_4_]^+^, [M + Na]^+^), monitored during the HPLC-QTOF-MS analysis of samples prepared by lyophilisation, and by solid phase extraction Strata-X.

The RSD of the combined peak areas of all monitored ions in chloramphenicol was found to be 30% during the analysis of samples prepared using lyophilisation, compared to 4% during samples prepared using Strata-X. This indicated that the Strata-X method of sample preparation produced more stability in adduct formation. A similar trend was seen, though to a lesser extent, in the combined peak areas of monitored ions formed by valinomycin. During the analysis of samples prepared by lyophilisation the RSD of the valinomycin external standard was found to be 39%, whereas it was 22% during the analysis of samples prepared with Strata-X (Figure 2). The variation in the individual adducts of the lyophilisation samples were even more pronounced, particularly with the sodium adduct, which initially made up 87% of the total combined adduct area, and then dropped to 29% before then increasing to 67%. This dramatic change in total relative adduct composition was not seen in the samples from Strata-X preparation.

These results showed that sample preparation via lyophilisation resulted in significantly more instability in ion source conditions compared to SPE Strata-X and, therefore, the SPE method was chosen for quantification experiments.

### 2.2. Quantification

**Figure 3:**
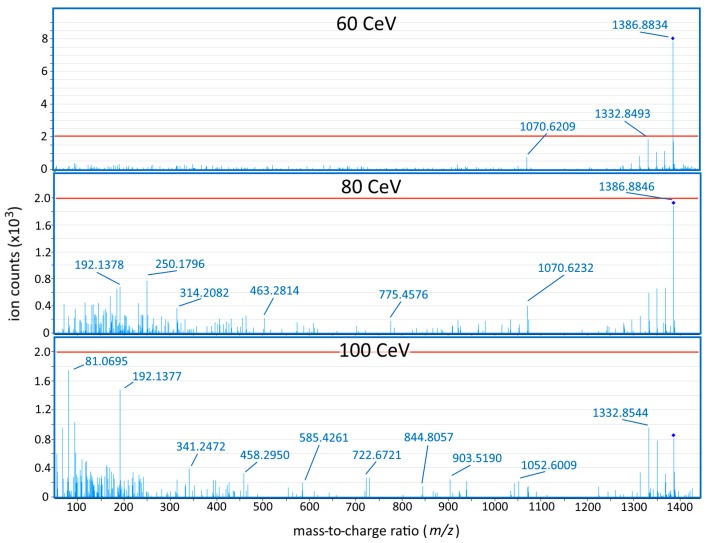
Fragmentation of karmitoxin at three different collision energies (60, 80, and 100 CeV) on the Agilent QTOF system. The red line indicates a 5% individual product ion yield. The average mass accuracy of the precursor ion in this dataset was 2.1 ppm. The ions with the highest individual product ion yields in the 60 and 80 CeV spectra were products of the consecutive neutral loss of water, and each had individual product ion yields <5%. The most prevalent non-water loss fragmentation ion observed between fragmentation spectra was the 1070.62 ion, likely arising from the loss of the apolar arm with a resulting molecular formula of C_55_H_91_NO_19_.

The fragmentation of karmitoxin was investigated on an Agilent HPLC-QTOF system. This system was utilized to investigate the fragmentation of karmitoxin due to its transferability of ion source parameters to the available Agilent UHPLC-QqQ for potential method transfer. The MS/MS fragmentation of karmitoxin showed very limited fragmentation occurring from 10 to 60 CeV, while above 60 CeV extensive fragmentation was observed, with both (i) very poor product ion yields (<5%) (Figure 3), and (ii) not very specific fragment ions (water loss ions), the most significant of which was the [M − 3(H_2_O) + H]^+^ fragmentation ion with a 4.8% yield at 60 CeV (Figure 3). In most MS/MS spectrum with the greatest individual product ion yields, due to poor fragmentation, the precursor ion was the most abundant ion. Evaluation of the signal-to-noise ratio (S/N) in six samples by MS/MS on an Agilent UHPLC-QqQ instrument, using this transition in comparison to the protonated adduct on the Bruker HPLC-QTOF-MS system, showed an average 7.6-fold lower S/N, leading to the continued use of the high-resolution instrument. The Bruker HPLC-QTOF-MS was utilized throughout the remainder of the study due to more favourable ionization and adduct formation.

**Figure 4:**
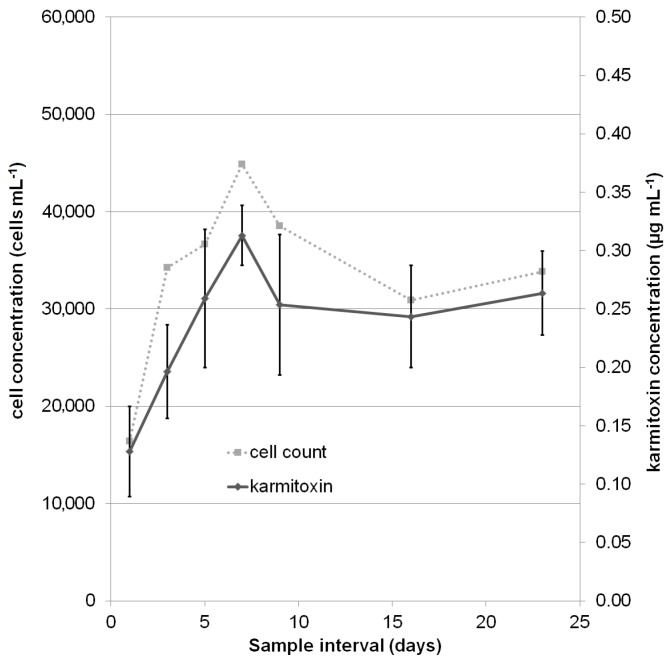
*K. armiger* culture grown in ammonium-substituted f/2 media: a comparison of culture cell concentration (cells·mL^−1^) to the concentration of karmitoxin (μg·mL^−1^). The concentration of karmitoxin correlated with cell concentration, a linear regression of karmitoxin per cell had an R^2^ value of 0.84 across all growth phases (Figure S2). The displayed standard deviation for each sample day is the standard deviation across all of the biological replicates. The average relative standard deviation across all sample points was 20%.

**Figure 5:**
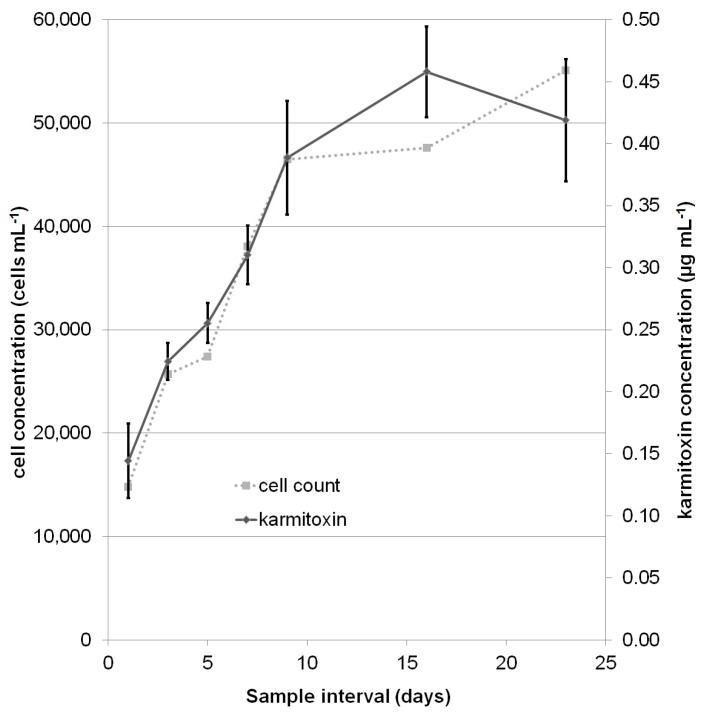
*K. armiger* culture grown in f/2 media in the presence of *Rhodomonas salina* as a food source: a comparison of cell concentration (cells·mL^−1^) to the concentration of karmitoxin (μg·mL^−1^). As with the cultures grown on ammonium-substituted f/2 media, the concentration of karmitoxin correlated with cell concentration, a linear regression of karmitoxin per cell had an R^2^ value of 0.93 across all growth phases (Figure S3). The stationary growth phase appeared to begin at day 8, two days later than that of *K. armiger* grown in ammonium-substituted f/2 media. The displayed standard deviation for each sample day is the standard deviation across all of the biological replicates. The average relative standard deviation across all biological replicates was 10%.

The concentration of karmitoxin in laboratory grown cultures was assessed over 23 days, up to and including the stationary phase, to determine the relationship between karmitoxin and the growth phase (Figure 4 and Figure 5) and to determine the effect of mixotrophy on karmitoxin production (Figure 5). The samples taken were quantified against a calibration curve of karmitoxin in methanol (MeOH) and values adjusted based on a two-day calculated recovery from the standard addition to ^13^C-labelled culture samples. The calculated apparent recovery was 57% with a RSD of 20%. The high RSD was mostly influenced by the lowest level (level 1: 0.06 μg·mL^−1^). Excluding the lowest level from the calculation resulted in a RSD of 9.3%, however, as two of the 126 samples had concentrations which fell between level 1 and level 2, all levels were included in calculations. The calibration curve had linearity with an R^2^ value of 0.972 (Figure S1). Despite testing a range of different stationary phases, and utilizing the solid substrate with the greatest relative recovery in method development, recovery was still low, albeit reproducible. A definitive cause of the low recovery cannot be made, however, this class of metabolites has previously been described as being “sticky” [57] with the ability to adhere to a range of substrates including apolar polytetrafluoroethylene (PTFE) filters and polar glass fibre filters [19]. These unusual characteristics can perhaps be attributed to the intramolecular polarity variation of these metabolites, having both significant polar and apolar regions.

Across all growth phases there was a proportional relationship between karmitoxin concentration and cell count in both growth mediums (Figures S2 and S3). The average concentration of karmitoxin in *Rhodomonas salina* fed *K. armiger* was 8.8 pg·cell^−1^ and had a RSD of 9.1%. The average concentration of karmitoxin for *K. armiger* grown in ammonium substituted f/2 media was 7.1 pg·cell^−1^ and had a RSD of 11%. Using ANOVA statistical analysis it was determined that this difference in karmitoxin production between the two growth media was statistically significant (*p* < 0.0001) (Table S2), however, it is possible that this observed 23% increase in karmitoxin concentration in mixotrophic cultures is associated with increased nutrition from *R. salina* rather than being a predator-prey induced phenomenon. This proportional production of karmitoxin in relationship to cell concentration seen here with *K. armiger* is in contrast to a semi-quantification study on another ichthyotoxic microalgae, the haptophyte *Prymnesium parvum*, in relation to the ichthyotoxins prymnesin 1 and 2, which found that the concentration of these large polyketides was not directly proportional to cell concentration [20].

Using Strata-X for sample preparation, the matrix effect was very low (106%, RSD 13%). The limit of quantification (LOQ) was found to be 0.11 μg·mL^−1^, and the limit of detection (LOD) was 0.03 μg·mL^−1^. This method could be adjusted to obtain a lower LOQ, such as increasing sample concentration, however for the purposes of this study, a lower LOQ was not necessary with cell concentrations above 10,000 cell·mL^−1^.

## 3. Materials and Methods

### 3.1. Chemicals, Standards and Materials

All solvents used for sample preparation were of HPLC grade and purchased from Sigma-Aldrich (Schnelldorf, Germany). All solvents used for sample analysis, water (H_2_O), methanol (MeOH), and acetonitrile (ACN), were of LCMS grade and purchased from Sigma-Aldrich. Formic acid (≥96%) was purchased from Thermo Fisher Scientific (Waltham, MA, USA). H_2_O for SPE was purified on a Milli-Q system (Merck Millipore, Billerica, MA, USA). Iturin A2 (>95%) was purchased from Sigma-Aldrich and the stock solution (20 μg·mL^−1^) was prepared in HPLC grade MeOH. Sodium bicarbonate (NaHCO_3_), 98% ^13^C-labelled, was purchased from Sigma-Aldrich. Fluorescence derivatisation was achieved with AccQ-Fluor reagent WAT052880 (Waters; Milford, MA, USA). The mixture of fumonisin B_1_ (49.9 μg·mL^−1^) and fumonisin B_2_ (50.6 μg·mL^−1^) in H_2_O/ACN (1:1, *v*/*v*) was purchased from Romer Labs (Tulln, Austria). External standards valinomycin and chloramphenicol were purchased from Sigma-Aldrich. Five different, 3 mL, 30 mg, SPE cartridges, Strata-X, Strata-SCX, Strata-WCX, Strata-SAX, Strata-MAX, were purchased from Phenomenex (Torrance, CA, USA). Three millilitre, 30 mg Oasis-HLB SPE cartridges were purchased from Waters.

### 3.2. Algal Cultures

The *K. armiger*, strain K-0668, and *R. salina*, strain K-1487, were acquired from the Scandinavian Culture Collection for Algae and Protozoa (Marine Biological Section, University of Copenhagen, Copenhagen, Denmark). The *R. salina* cultures were maintained in f/2 media and *K. armiger* cultures were maintained in modified f/2 media with NO_3_^−^ substituted with 50 μM NH_4_^+^ both prepared using pasteurised (95 °C, 1.5 h) seawater at 30 practical salinity units with aeration and a light-dark cycle of 14:10 h. NaHCO_3_ was added to media, at a final concentration of 0.5 mM, to counter losses from the heat treatment of the seawater. Aeration was supplied to avoid elevated pH in the cultures due to high rates of photosynthesis. Cultures were grown at a constant temperature of 15 °C with an irradiance of 150 μmol photons m^−2^·s^−1^. Cultures with ^13^C enrichment were grown under the same conditions with the exception of the carbon source and aeration. In ^13^C-enriched cultures, aeration was removed and a total of 2.6 mM ^13^C-labelled NaHCO_3_ was added in multiple steps to achieve 52% ^13^C incorporation (determined by HPLC-QTOF-MS). In the absence of aeration, the pH of ^13^C-enriched cultures was adjusted as needed with 1 M HCl solution. Cultures utilized in karmitoxin standard preparation were harvested during the stationary phase, which was determined by cell count. Cell densities were determined during growth by light microscope.

### 3.3. Karmitoxin Standard Quantification

Multiple cultures of *K. armiger* (total 80 L) were extracted utilizing a method previously described [30], in order to acquire a purified sample of karmitoxin to be used for quantification. The karmitoxin standard was compared in accurate mass (<2 ppm) and retention time to a previously-isolated and structurally-elucidated (by nuclear magnetic resonance spectroscopy) sample [30], which was obtained using the same purification protocol.

The concentration of the isolated toxin was determined by an average of two external calibration curves using a certified reference standard mixture of fumonisin B_1_ and fumonisin B_2_ (both containing a primary amine, as with karmitoxin), derivatised with AccQ-Fluor reagent. Fumonisin mixture, 20 μL, was combined with 140 μL borate buffer in a 300 μL glass vial. The solution was briefly vortexed and 40 μL of reconstituted AccQ-Fluor reagent (3.0 μg·mL^−1^, in ACN) was added to the vial, which was homogenised immediately and placed on a heating block at 55 °C for 10 min, resulting in a derivatised fumonisin stock solution (4.99 μg·mL^−1^). Five levels of the stock solution were prepared in triplicate, applying serial dilutions with borate buffer resulting in the following concentrations: 0.07, 0.15, 0.31, 0.62, and 1.25 μg·mL^−1^. Using an equivalent derivatisation procedure, 10 μL of karmitoxin stock solution was derivatised in four replicates. These samples were submitted to HPLC-fluorescence analysis, with 5 μL injection volumes (Figures S4 and S5).

Derivatised samples were analysed on an Ultimate 3000 UHPLC system (Dionex; Sunnyvale, CA, USA) coupled to an Agilent 1100 Fluorescence detector (Agilent Technologies; Santa Clara, CA, USA) operating in *λ* excitation 250 nm and emission 395 nm. Chromatographic separation was achieved on a Phenomenex Kinetex reversed phase column (2.6 μm, C_18_, 100 Å, 100 mm × 2.1 mm). A linear gradient elution was used for separation at a constant flow rate of 400 μL·min^−1^, using two eluents, eluent A: H_2_O, and eluent B: ACN, both of which contained 20 mM formic acid. The gradient started at 10% B and increased to 80% B over 12 min, then to 100% B over 0.5 min, and was held at 100% B for 1.5 min before being re-equilibrated for subsequent injections.

### 3.4. Culture Sampling

To determine the best method for sampling preparation, nine different culture sampling methods were assessed; whole culture drying (detailed in (1) below), liquid/liquid extraction (2), lyophilisation (3), and six different SPE substrates (4). For each method a subsample (3 mL) from a larger *K. armiger* culture (1 L) in the exponential growth phase, determined by cell concentration derived from light microscope counts, was extracted.

(1) The culture subsample (3 mL) was directly dried under a N_2_ stream (25 °C). The dried sample was dissolved in MeOH (1 mL) and placed in an ultrasonic bath (5 min). A subsample (800 μL) was transferred to an Eppendorf tube (1.5 mL) and centrifuged (10 min, 9000 RCF). The supernatant (500 μL) was transferred to a glass HPLC vial for analysis by LC-MS. The method was performed in five replicates.
(2) Ethlyacetate (EtOAc) (3 mL) was added to the culture subsample (3 mL) and placed in an ultrasonic bath (5 min). The EtOAc layer (2.8 mL) was dried under a N_2_ stream (25 °C). The dried sample was dissolved in MeOH (1 mL) and placed in an ultrasonic bath (5 min). A subsample (800 μL) was transferred to an Eppendorf tube (1.5 mL) and centrifuged (10 min, 9000 RCF). The supernatant (500 μL) was transferred to a glass HPLC vial for analysis by LC-MS. The method was performed in five replicates.
(3) The culture subsample (3 mL) was frozen to −80 °C and then lyophilised. The lyophilised sample was dissolved in MeOH (2 mL) and placed in an ultrasonic bath (5 min). A subsample (800 μL) was transferred to an Eppendorf tube (1.5 mL) and centrifuged (10 min, 9000 RCF). The supernatant (500 μL) was transferred to a glass HPLC vial for analysis by LC-MS. The method was performed in five replicates.
(4) The culture subsample (3 mL) was directly loaded onto a conditioned (MeOH, 2 mL) and equilibrated (Milli-Q H_2_O, 2 mL) SPE cartridge (30 mg). This was followed by a salt removal washing step (H_2_O, 3 mL) and flushing with air. Sample collection then followed with elution (MeOH, 1 mL) and a final flushing with air. A subsample (500 μL) was transferred to a glass HPLC vial for analysis. The method was performed in triplicates on six different solid phases: Strata-X, Strata-SCX, Strata-WCX, Strata-SAX, Strata-MAX, and Oasis-HLB.

Samples from each of the methods were submitted to HPLC-QTOF-MS analysis, using 2 μL injection volumes. The two most effective sampling methods, lyophilisation and Strata-X SPE, were investigated for their robustness over extended sample sequences and multiple injections to determine their appropriateness for quantitative experiments, especially targeting fouling on the ion source by salts. A 1 L culture of *K. armiger* was grown in triplicate, in ammonium-replaced f/2 media. Six 3 mL culture samples were taken from each of the cultures at specific time intervals (0, 2, 4, 8, 12, and 24 h). For each of the time intervals, half of the culture samples were prepared using one of the two sample preparation methods.

Samples from one method were analysed separately from the other on consecutive days. Prior to the analysis of samples for each sample preparation procedure the source of the MS was thoroughly cleaned. Source conditions, in regards to ionization repeatability, were monitored using external standards valinomycin and chloramphenicol, routinely injected as a mixture after every six sample injections. Multiple ions formed by each of the standards were monitored over time to determine changes in the proportion of adduct formation. These ions were chloramphenicol: 323.0196 ([M + H]^+^), 345.0046 ([M + Na]^+^), 509.9878 ([3M + Fe(II)]^2+^), 695.9481 ([2M − 2H + Fe(III)]^+^); and valinomycin: 1111.6384 ([M + H]^+^), 1128.6650 ([M + NH_4_]^+^), 1133.6204 ([M + Na]^+^). Ions of chloramphenicol were integrated with an *m*/*z* tolerance of 0.002 Da, and ions of valinomycin were integrated with an *m*/*z* tolerance of 0.005 Da, each resulting in a minimum mass accuracy of 5 ppm. The mixture of chloramphenicol and valinomycin was chosen as an external standard due to the two compounds’ range of characteristics: chloramphenicol, a small (323.13 g·mol^−1^) early-eluting (4.24 min), nitro-containing aromatic, and valinomycin, a large (1111.32 g·mol^−1^), late-eluting (11.64 min), macrocyclic peptide. Of the two methods SPE sample preparation with Strata-X was found to be superior; this method was selected for quantitative experiments.

### 3.5. Culture Quantification

Two treatments were applied to 1 L cultures of *K. armiger* in triplicate, one of the triplicates was grown with f/2 media and fed *R. salina*, and the other was grown in ammonium replaced f/2 media. Triplicate 3 mL culture samples were taken from each of the cultures at specific time intervals (1, 3, 5, 7, 9, 16, and 23 days), and using Strata-X SPE sample preparation. Iturin A2 was added to each of the samples as an internal standard. The samples for each of the methods were then submitted to HPLC-QTOF-MS for analysis, using 2 μL injection volumes.

The concentration of karmitoxin in the samples from the experimental cultures was quantified using a non-weighted linear external calibration performed in triplicate at seven levels. The stock solution of karmitoxin (19.40 μg·mL^−1^, determined by AccQ-Flour derivatisation) was diluted to appropriate concentrations in MeOH together with the internal standard, iturin A2. This resulted in an external calibration curve of karmitoxin with the following concentrations: 0.00, 0.06, 0.12, 0.24, 0.60, and 0.80 μg·mL^−1^. LOD was calculated as the standard error divided by the correlation coefficient multiplied by 3 and the LOQ was calculated as the standard error divided by the correlation coefficient multiplied by 10. Recovery was determined on two days by spiking karmitoxin in Milli-Q H_2_O before the SPE sample preparation procedure at six levels (0.00, 0.06, 0.12, 0.24, 0.60, and 0.80 μg·mL^−1^) in triplicate. Matrix effects were determined by spiking ^13^C incorporated cultures of *K. armiger* with karmitoxin after the sample preparation procedure, also in triplicate at the same six levels. There was no detection of unlabelled karmitoxin within ^13^C-labelled culture samples with no spiked karmitoxin. Recovery and matrix effects were determined in relation to the internal standard iturin A2. Integrated peak area of iturin A2 was calculated as a sum of three adducts (*m*/*z* 1043.5520, [M + H]^+^; *m*/*z* 1065.5340, [M + Na]^+^; *m*/*z* 522.2797, [M + 2H]^2+^) and the integrated peak area of karmitoxin was calculated as the sum of three adducts (*m*/*z* 1386.8872, [M + H]^+^; *m*/*z* 1387.8906, ^13^C-[M + H]^+^; *m*/*z* 704.9382, [M + H + Na]^2+^). Integration of all selected ions was achieved with a mass accuracy of <5 ppm, using ion specific *m*/*z* tolerances. Iturin A2 was selected as an internal standard for its similarity in monoisotopic ion mass and retention time to karmitoxin, *m*/*z* of 1043.5520 and 1386.8872, and retention times of 5.70 and 5.65 min, respectively (Figure S6).

### 3.6. Liquid Chromatography—High Resolution Mass Spectrometry

Culture samples were analysed by HPLC-QTOF-MS. A Dionex Ultimate 3000 with a diode array detector (DAD) equipped with a Phenomenex Kinetex column (2.6 μm, C_18_, 100 Å, 100 mm × 2.1 mm) was used for chromatographic separation and maintained at 40 °C. Two eluents were used: eluent A (H_2_O, 20 mM formic acid), and eluent B (ACN, 20 mM formic acid). Eluents were used in a gradient starting with 10–70% eluent B over 6.7 min, followed by 70–100% eluent B over 1.3 min, then held at 100% eluent B for 3 min. All analysis was carried out at a constant flow rate of 400 μL·min^−1^.

The HPLC system was coupled to a Maxis QTOF MS with electrospray ionisation (ESI) (Bruker Daltonics, Bremen, Germany). Analysis was carried out in positive ESI. The scan range was *m*/*z* 300–2500 with two scans per second. The capillary voltage was set to 4500 V, the nebuliser gas was at 1.8 bar, and the dry gas was 200 °C at a flow rate of 10 L·min^−1^.

Prior to the analysis of samples, the QTOF-MS was calibrated using a sodium formate calibrant with an accepted calibration being <1 ppm. In addition, all data files were recalibrated with an internal standard of sodium formate injected prior to initial sample elution for each sample.

### 3.7. Liquid Chromatography—Tandem Mass Spectrometry

The fragmentation of karmitoxin was investigated for potential quantification by SRM, using an Agilent Infinity 1290 UHPLC system (Agilent Technologies, Santa Clara, CA, USA) coupled to an Agilent 6545 QTOF MS and a similar UHPLC coupled to an Agilent 6490 Triple QqQ MS. A range of collision energies were tested (20, 40, 50, 60, 80, 100, and 150 CeV) to determine the optimal fragment ion yields.

## 4. Conclusions

Lyophilisation initially resulted in the lowest standard deviation and greatest intensity of all tested sample preparation methods. Over long injections sequences, however, samples prepared using this procedure were found to have compromised adduct formation stability. When applying the same injection sequence to SPE Strata-X prepared samples it was found to have a much more stable adduct formation and is therefore more suitable for quantification.

In a semi-quantitative analysis of the SPE sample preparation methods, based on the integrated peak areas, it appeared that all SPE methods had either similar or lower recoveries compared to the Strata-X stationary phase, assuming similar matrix effects. Although the apparent recovery of 57% for Strata-X is low, it was importantly found to be reproducible, particularly above the lowest spiked level, with a RSD of recovery for these levels of 9.3%. The related metabolites, the karlotoxins, have been described as “sticky” compounds [57], with the ability to be retained on many different substrates [19]. It is possible that these unusual properties of this family of metabolites contributed to the low recovery seen in our sample preparation procedure. 

Analysis of the fragmentation of karmitoxin for QqQ SRM quantification found that this metabolite had very poor fragmentation characteristics, producing low individual product ion yields and nonspecific fragmentation ions. This is thought to be due to this metabolites long, flexible, uninterrupted carbon backbone, and may be a characteristic of other amphidinol-like metabolites. Analysis of a few samples found that QTOF-based analysis provided higher S/N sensitivity compared to QqQ SRM analysis, due to these poor fragmentation characteristics. Altogether this report represents the first HRMS quantitative recovery experiment on a metabolite of the karlotoxin/amphidinol family of compounds, a group of more than 40 structurally-related microalgae metabolites (Table S1).

## Supplementary Materials

The following are available online at www.mdpi.com/1660-3397/15/9/278/s1. Figure S1: Calibration curve of karmitoxin in methanol; Figure S2: Relationship between karmitoxin and phototrophic cell density; Figure S3: Relationship between karmitoxin and mixotrophic cell density; Figure S4: Calibration curve of fumonisin B_1_; Figure S5: Calibration curve of fumonisin B_2_; Figure S6: Strata-X prepared sample with Bruker QTOF analysis; Table S1: Details of the amphidinol-like metabolites from dinoflagellates; Table S2: Concentration of karmitoxin per cell.
