# Supplementary material for: HPLC-HRMS Quantification of the Ichthyotoxin Karmitoxin from Karlodinium armiger
Source: Mar Drugs. 2017 Aug 31;15(9):278. doi: 10.3390/md15090278 (PMC5618417; doi:10.3390/md15090278)
Supplement: Supplementary file 1 [file marinedrugs-15-00278-s001.pdf]

Supporting Information for

HPLC-HRMS Quantification of the Ichthyotoxin Karmitoxin from *Karlodinium armiger*

Aaron John Christian Andersen,[1,2] Lívia Soman de Medeiros,[3] Sofie Bjørnholt Binzer,[4] Silas Anselm Rasmussen,[2] Per Juel Hansen,[4] Kristian Fog Nielsen,[2] Kevin Jørgensen,[1] and Thomas Ostenfeld Larsen [2]

[1] National Food Institute, Technical University of Denmark, Kemitorvet Building 202, 2800 Lyngby, Denmark

[2] Department of Biotechnology and Biomedicine, Technical University of Denmark, Building 221, 2800 Lyngby, Denmark

[3] Departamento de Química, Universidade Federal de São Paulo (UNIFESP), Rua São Nicolau, 210, CEP 09913-030, Diadema-SP, Brazil

[4] Marine Biological Section, Department of Biology, University of Copenhagen, Strandpromenaden 5, 3000 Helsingør, Denmark

Correspondence to: Thomas Ostenfeld Larsen (tol@bio.dtu.dk)

Table of Contents

Figure S1: *Calibration curve of karmitoxin in methanol* 2

Figure S2: *Relationship between karmitoxin and phototrophic cell density* 3

Figure S3: *Relationship between karmitoxin and mixotrophic cell density* 4

Figure S4: *Calibration curve of fumonisin B_1* 5

Figure S5: *Calibration curve of fumonisin B_2* 6

Figure S6: *Strata-X prepared sample with Bruker QTOF analysis* 7

Table S1: *Details of the amphidinol-like metabolites from dinoflagellates* 10

Table S2: *Concentration of karmitoxin per cell* 11

References for Supporting Information 12

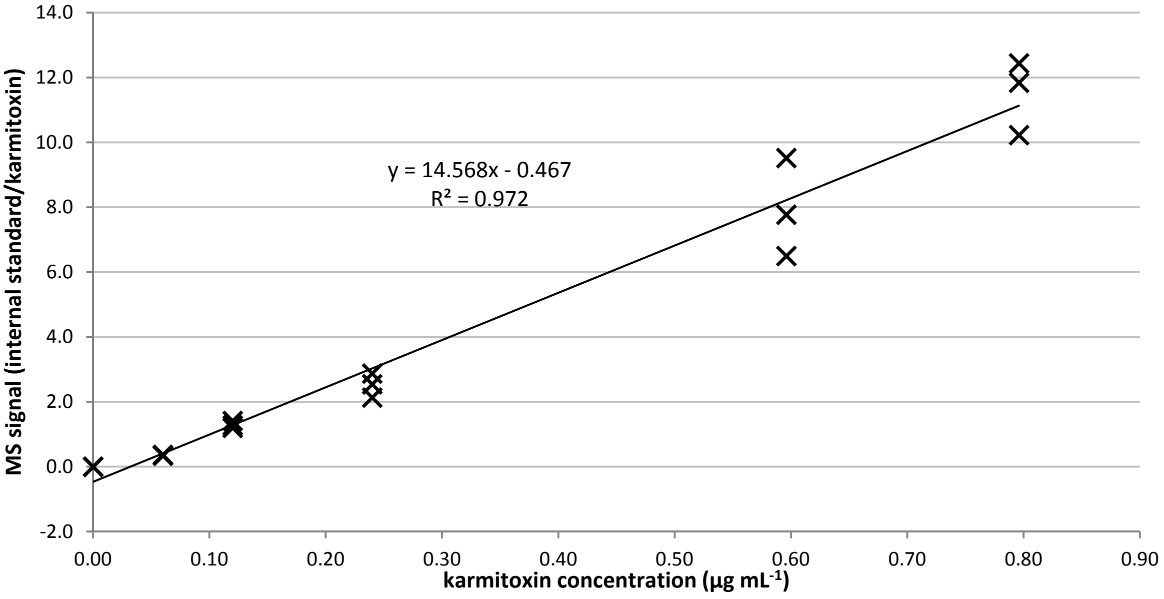

Figure S1: *Calibration curve of karmitoxin in methanol*

Calibration curve used for the quantification of karmitoxin in experimental cultures

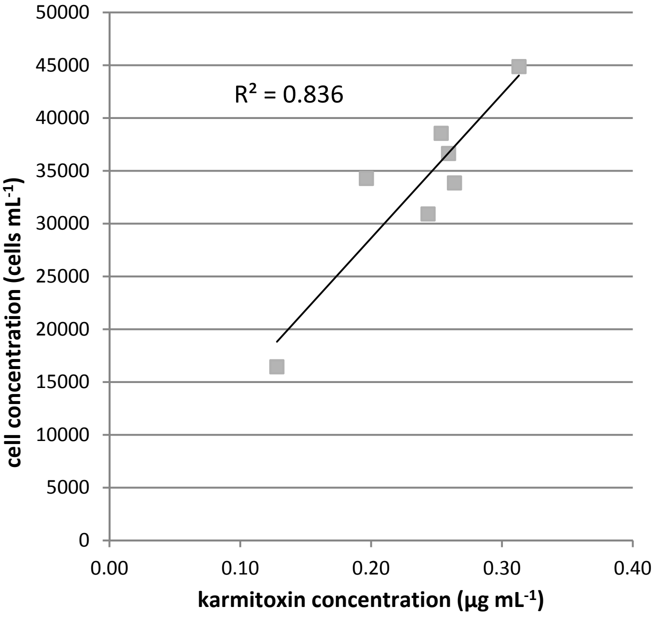

Figure S2: *Relationship between karmitoxin and phototrophic cell density*

Karmitoxin concentration compared to cell concentration in cultures grown in ammonium substituted f/2 media.

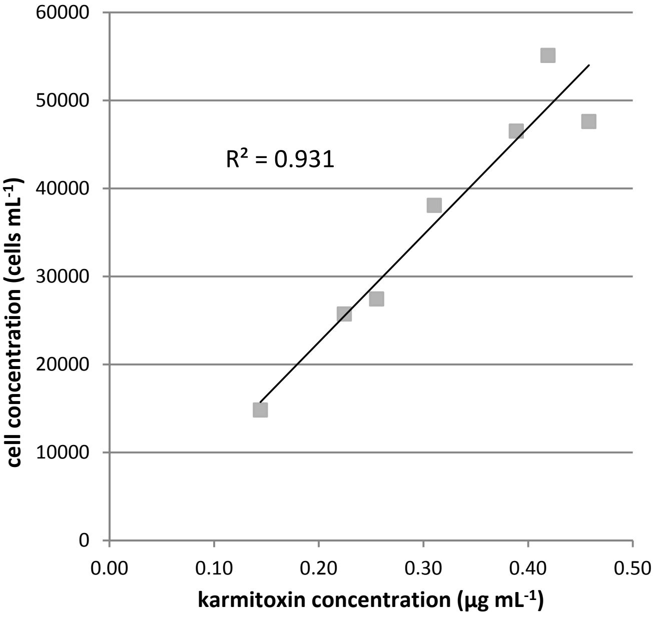

Figure S3: *Relationship between karmitoxin and mixotrophic cell density*

Karmitoxin concentration compared to cell count in cultures grown in f/2 media and fed *Rhodomonas salina*.

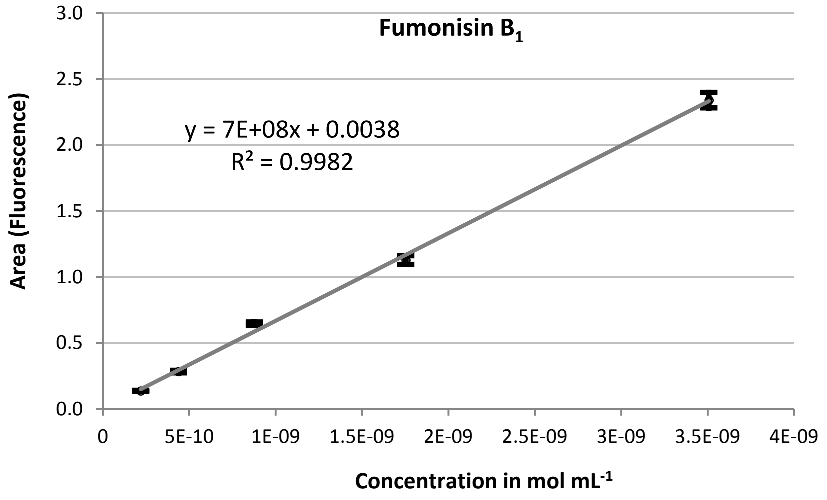

Figure S4: *Calibration curve of fumonisin* B_1

Calibration curves used for the quantification of karmitoxin standard.

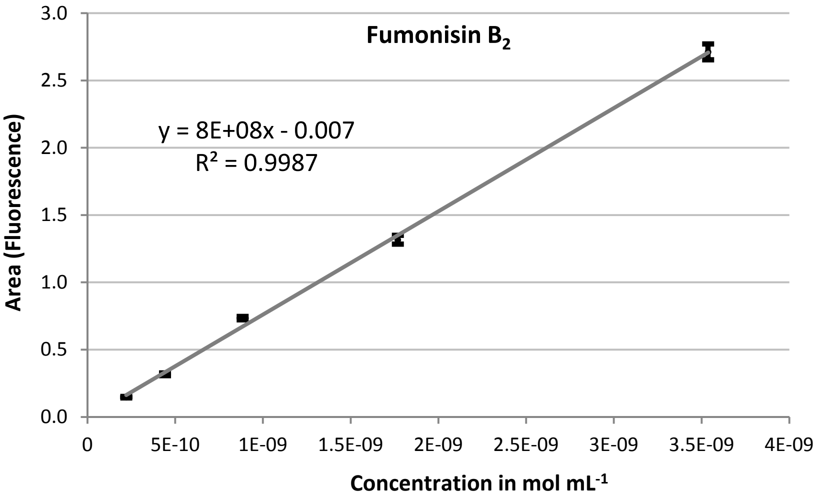

Figure S5: *Calibration curve of fumonisin B_2*

Calibration curves used for the quantification of karmitoxin standard.

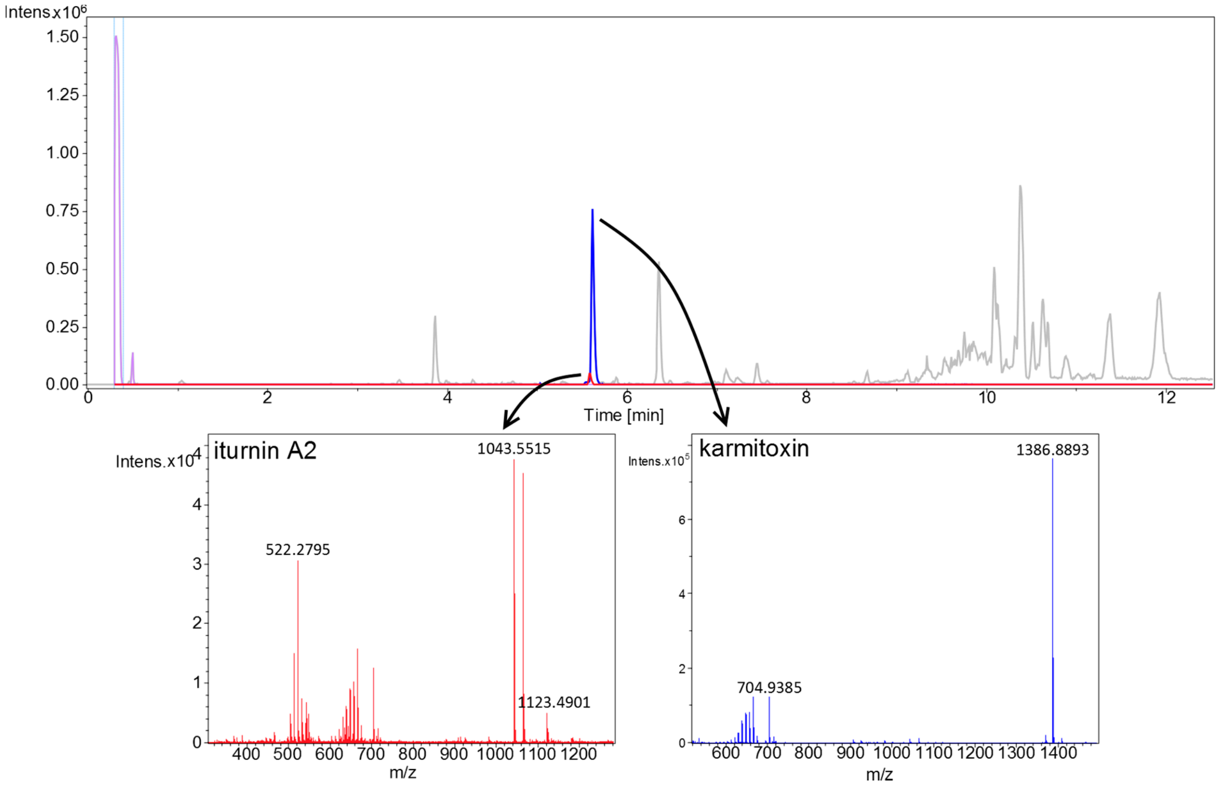

Figure S6: *Strata-X prepared sample with Bruker QTOF analysis*

This figure illustrates an example base peak chromatogram (grey line) of a Strata-X SPE prepared sample of *K. armiger* with an internal standard iturin A2 (indicated by the red extracted ion chromatogram). The blue extracted ion chromatogram indicates the $[M+H]^+$ ion of karmitoxin, and the purple extracted ion chromatogram indicates the internal calibrant.

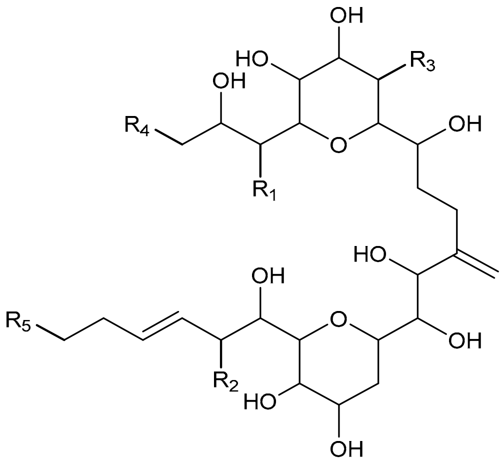

Name	Molecular Formula	Monoisotopic Mass	Source	R_1	R_2	R_3	R_4 (polar arm) Formula	backbone	methyl	R_5 (non-polar arm) Formula	backbone	triene	Reference
Karlotoxin 9	$C_{65}H_{117}ClO_{24}$	1316.7623	*Karlodinium sp.*	H	OH	OH	$C_{34}H_{67}O_{12}$	C_{31}	3	$C_{14}H_{22}ClO_2$	C_{14}	No	(Waters et al. 2015)
Karlotoxin 8	$C_{65}H_{117}ClO_{24}$	1316.7623	*Karlodinium sp.*	H	OH	OH	$C_{34}H_{67}O_{12}$	C_{31}	3	$C_{16}H_{26}ClO_2$	C_{16}	No	(Waters et al. 2015)
Symbiopolyol	$C_{60}H_{100}NaO_{23}S$	1243.6274	*Amphidinium sp.*	OH	OH	H	$C_{27}H_{49}O_{12}S$	C_{25}	2	$C_{16}H_{23}O_2$	C_{16}	Yes	(Hanif et al. 2010)
Amphidinol 17	C63H110O24S	1282.7108	*Amphidinium carterae*	OH	OH	H	$C_{30}H_{59}O_{13}S$	C_{27}	3	$C_{16}H_{23}O_2$	C_{16}	Yes	(Meng et al. 2010)
Amphidinol 11	$C_{71}H_{122}NaO_{28}S$	1477.7741	*Amphidinium carterae*	OH	OH	H	$C_{38}H_{71}O_{17}S$	C_{33}	5	$C_{16}H_{23}O_2$	C_{16}	Yes	(Echigoya et al. 2005)
Lingshuiol B	$C_{60}H_{100}NaO_{23}S$	1243.6274	*Amphidinium sp.*	OH	OH	H	$C_{27}H_{49}O_{12}S$	C_{25}	2	$C_{16}H_{23}O_2$	C_{16}	Yes	(Huang et al. 2004)
Carteraol E	$C_{74}H_{126}O_{24}$	1398.8639	*Amphidinium carterae*	OH	OH	H	$C_{41}H_{75}O_{13}$	C_{37}	4	$C_{16}H_{23}O_2$	C_{16}	Yes	(Huang et al. 2009)
Amphidinol 7	$C_{59}H_{100}O_{23}S$	1208.6376	*Amphidinium klebsii*	OH	OH	H	$C_{26}H_{49}O_{12}S$	C_{23}	3	$C_{16}H_{23}O_2$	C_{16}	Yes	(Morsy et al. 2005)
Amphidinol 12	$C_{68}H_{116}O_{26}S$	1380.7476	*Amphidinium carterae*	OH	OH	H	$C_{35}H_{65}O_{15}S$	C_{33}	2	$C_{16}H_{23}O_2$	C_{16}	Yes	(Echigoya et al. 2005)
Amphidinol 2	$C_{71}H_{122}O_{25}$	1374.8275	*Amphidinium klebsii*	OH	OH	H	$C_{38}H_{71}O_{14}$	C_{33}	5	$C_{16}H_{23}O_2$	C_{16}	Yes	(Paul et al. 1995)
Amphidinol 4	$C_{68}H_{116}O_{23}$	1300.7907	*Amphidinium carterae and A. klebsii*	OH	OH	H	$C_{35}H_{65}O_{12}$	C_{33}	2	$C_{16}H_{23}O_2$	C_{16}	Yes	(Houdai et al. 2001)

Name	Molecular Formula	Monoisotopic Mass	Source	R_1	R_2	R_3	R_4 (polar arm)			R_5 (non-polar arm)			Reference
							Formula	backbone	methyl	Formula	backbone	triene	
Amphidinol 6	$C_{70}H_{120}O_{24}$	1344.8170	*Amphidinium klebsii*	OH	OH	H	$C_{37}H_{69}O_{13}$	C_{35}	2	$C_{16}H_{23}O_2$	C_{16}	Yes	(Paul et al. 1997)
Amphidinol 10	$C_{66}H_{112}O_{23}$	1272.7594	*Amphidinium spp.*	OH	OH	H	$C_{33}H_{61}O_{12}$	C_{31}	2	$C_{16}H_{23}O_2$	C_{16}	Yes	(Echigoya et al. 2005)
Lingshuiol A	$C_{66}H_{112}O_{23}$	1272.7594	*Amphidinium sp.*	OH	OH	H	$C_{33}H_{61}O_{12}$	C_{31}	2	$C_{16}H_{23}O_2$	C_{16}	Yes	(Huang et al. 2004)
Amdigenol G	$C_{60}H_{102}NaO_{25}S$	1277.6329	*Amphidinium sp.*	OH	OH	H	$C_{27}H_{49}O_{12}S$	C_{25}	2	$C_{16}H_{25}O_4$	C_{16}	No	(Inuzuka et al. 2014)
Amdigenol A	$C_{104}H_{178}NaO_{43}S$	2170.1360	*Amphidinium sp*	OH	OH	H	$C_{71}H_{125}O_{30}S$	C_{66}	5	$C_{16}H_{25}O_4$	C_{16}	No	(Inuzuka et al. 2012)
Amphidinol 14	$C_{59}H_{102}NaO_{25}S$	1265.6329	*Amphidinium klebsii*	OH	OH	H	$C_{26}H_{49}O_{12}S$	C_{23}	3	$C_{16}H_{25}O_4$	C_{16}	Yes	(Morsy et al. 2006)
Luteophanol A	$C_{60}H_{102}O_{25}S$	1254.6431	*Amphidinium sp.*	OH	H	H	$C_{27}H_{49}O_{12}S$	C_{25}	2	$C_{16}H_{25}O_4$	C_{16}	No	(Doi et al. 1997)
Luteophanol D	$C_{67}H_{116}O_{25}$	1320.7806	*Amphidinium sp.*	OH	OH	H	$C_{34}H_{63}O_{12}$	C_{32}	2	$C_{16}H_{25}O_4$	C_{16}	No	(Kubota et al. 2005)
Amphidinol 15	$C_{59}H_{102}O_{22}$	1162.6863	*Amphidinium klebsii*	OH	OH	H	$C_{26}H_{49}O_9$	C_{23}	3	$C_{16}H_{25}O_4$	C_{16}	Yes	(Morsy et al. 2006)
Karlotoxin 2	$C_{67}H_{121}ClO_{24}$	1344.7936	*Karlodinium veneficum*	H	OH	OH	$C_{34}H_{67}O_{12}$	C_{31}	3	$C_{16}H_{26}ClO_2$	C_{16}	No	(Peng et al. 2010)
4,5-dihydro-karlotoxin 2	$C_{67}H_{123}ClO_{24}$	1346.8093	*Karlodinium veneficum*	H	OH	OH	$C_{34}H_{69}O_{12}$	C_{31}	3	$C_{16}H_{26}ClO_2$	C_{16}	No	(Cai et al. 2016)
4,5-dihydro-dechloro-karlotoxin 2	$C_{67}H_{124}O_{24}$	1312.8483	*Karlodinium veneficum*	H	OH	OH	$C_{34}H_{69}O_{12}$	C_{31}	3	$C_{16}H_{27}O_2$	C_{16}	No	(Cai et al. 2016)
Karatungiol B	$C_{73}H_{130}O_{27}$	1438.8799	*Amphidinium sp.*	OH	OH	OH	$C_{40}H_{73}O_{15}$	C_{37}	3	$C_{16}H_{29}O_2$	C_{16}	No	(Washida et al. 2006)
Lingshuiol	$C_{69}H_{122}O_{25}$	1350.8275	*Amphidinium sp.*	OH	OH	H	$C_{36}H_{65}O_{14}$	C_{33}	3	$C_{16}H_{29}O_2$	C_{16}	No	(Huang et al. 2004)
Karatungiol A	$C_{73}H_{132}O_{28}$	1456.8905	*Amphidinium sp.*	OH	OH	OH	$C_{40}H_{75}O_{16}$	C_{37}	3	$C_{16}H_{29}O_2$	C_{16}	No	(Washida et al. 2006)
Luteophanol C	$C_{67}H_{116}O_{25}$	1320.7806	*Amphidinium sp.*	OH	OH	H	$C_{33}H_{61}O_{12}$	C_{31}	2	$C_{17}H_{27}O_4$	C_{17}	No	(Kubota et al. 1998)
Luteophanol B	$C_{67}H_{116}O_{25}$	1320.7806	*Amphidinium sp.*	OH	OH	H	$C_{33}H_{61}O_{12}$	C_{31}	2	$C_{17}H_{27}O_4$	C_{17}	No	(Kubota et al. 1998)
64E-Chlorokarlotoxin 3	$C_{68}H_{123}ClO_{24}$	1358.8093	*Karlodinium veneficum*	H	OH	OH	$C_{34}H_{67}O_{12}$	C_{31}	3	$C_{17}H_{28}ClO_2$	C_{17}	No	(Van Wagoner et al. 2010)
10-O-Sulfokarlotoxin 3	$C_{68}H_{124}O_{27}S$	1404.8051	*Karlodinium veneficum*	H	OH	OH	$C_{34}H_{67}O_{15}S$	C_{31}	3	$C_{17}H_{29}O_2$	C_{17}	No	(Van Wagoner et al. 2010)
Karlotoxin 3	$C_{68}H_{124}O_{24}$	1324.8483	*Karlodinium veneficum*	H	OH	OH	$C_{34}H_{67}O_{12}$	C_{31}	3	$C_{17}H_{29}O_2$	C_{17}	No	(Van Wagoner et al. 2010)

Name	Molecular Formula	Monoisotopic Mass	Source	R_1	R_2	R_3	R_4 (polar arm)			R_5 (non-polar arm)			Reference
							Formula	backbone	methyl	Formula	backbone	triene	
Amphidinol 19	$C_{71}H_{122}NaO_{27}S$	1461.7792	*Amphidinium carterae*	OH	OH	H	$C_{36}H_{69}O_{16}S$	C_{33}	3	$C_{18}H_{25}O_2$	C_{18}	Yes	(Nuzzo et al. 2014)
Amphidinol 13	$C_{70}H_{118}NaO_{26}S$	1429.7530	*Amphidinium carterae*	OH	OH	H	$C_{35}H_{65}O_{15}S$	C_{33}	2	$C_{18}H_{25}O_2$	C_{18}	Yes	(Echigoya et al. 2005)
Amphidinol (Amphidinol 1)	$C_{73}H_{126}O_{27}S$	1466.8207	*Amphidinium klebsii*	OH	OH	H	$C_{38}H_{73}O_{16}S$	C_{35}	3	$C_{18}H_{25}O_2$	C_{18}	Yes	(Satake et al. 1991)
Amphidinol 18	$C_{71}H_{122}O_{24}$	1358.8326	*Amphidinium carterae*	OH	OH	H	$C_{36}H_{69}O_{13}$	C_{33}	3	$C_{18}H_{25}O_2$	C_{18}	Yes	(Nuzzo et al. 2014)
Amphidinol 9	$C_{70}H_{118}O_{23}$	1326.8064	*Amphidinium carterae*	OH	OH	H	$C_{35}H_{65}O_{12}$	C_{33}	2	$C_{18}H_{25}O_2$	C_{18}	Yes	(Echigoya et al. 2005)
Amphidinol 5	$C_{72}H_{122}O_{24}$	1370.8326	*Amphidinium klebsii*	OH	OH	H	$C_{37}H_{69}O_{13}$	C_{35}	2	$C_{18}H_{25}O_2$	C_{18}	Yes	(Paul, et al. 1997)
Amphidinol 3	$C_{70}H_{118}O_{23}$	1326.8064	*Amphidinium klebsii*	OH	OH	H	$C_{35}H_{65}O_{12}$	C_{33}	2	$C_{18}H_{25}O_2$	C_{18}	Yes	(Murata et al. 2010)
65E-Chlorokarlotoxin 1	$C_{69}H_{125}ClO_{24}$	1372.8249	*Karlodinium veneficum*	H	OH	OH	$C_{34}H_{67}O_{12}$	C_{31}	3	$C_{18}H_{30}ClO_2$	C_{18}	No	(Van Wagoner et al. 2010)
karmitoxin	$C_{73}H_{127}NO_{23}$	1385.8799	*Karlodinium armiger*	H	OH	OH	$C_{38}H_{68}NO_{11}$	$C_{36}N$	2	$C_{18}H_{31}O_2$	C_{18}	No	(Rasmussen et al. 2017)
10-O-Sulfokarlotoxin 1	$C_{69}H_{126}O_{27}S$	1418.8207	*Karlodinium veneficum*	H	OH	OH	$C_{34}H_{67}O_{15}S$	C_{31}	3	$C_{18}H_{31}O_2$	C_{18}	No	(Van Wagoner et al. 2010)
Karlotoxin 1	$C_{69}H_{126}O_{24}$	1338.8639	*Karlodinium veneficum*	H	OH	OH	$C_{34}H_{67}O_{12}$	C_{31}	3	$C_{18}H_{31}O_2$	C_{18}	No	(Van Wagoner et al. 2008)
Ostreol A	$C_{67}H_{112}N_2O_{23}$	1312.7656	*Ostreopsis cf. ovata*	OH	OH	H	$C_{36}H_{61}N_2O_{12}$	$C_{34}N_2$	2	$C_{14}H_{23}O_2$	C_{14}	No	(Hwang et al. 2013)
2-desulfo-amphidinol 7	$C_{59}H_{100}O_{20}$	1128.6808	*Amphidinium klebsii*	OH	OH	H	$C_{26}H_{49}O_9$	C_{23}	3	$C_{16}H_{23}O_2$	C_{16}	Yes	(Morsy et al. 2006)
Amdigenol E*	$C_{82}H_{138}NaO_{37}S$	1769.8535	*Amphidinium sp.*	NA	NA	NA	NA	NA	-	$C_{16}H_{25}O_4$	C_{16}	No	(Inuzuka et al. 2014)
Prorocentrol*	$C_{68}H_{114}O_{34}$	1474.7192	*Prorocentrum hoffmannianum*	NA	NA	NA	$C_{31}H_{57}O_{17}$	C_{28}	3	$C_{22}H_{33}O_6$	C_{22}	Yes	(Sugahara et al. 2011)

Table S1: *Details of the amphidinol-like metabolites from dinoflagellates*

Details of the amphidinol-like metabolites from dinoflagellates, and compounds (2-desulfo-amphidinol 7) and metabolites (amdigenol E, prorocentrol) closely related to these. * = these metabolites do not share the common core structure, but do have structural similarities.

pg cell^{-1}	
Fed	NH_4^+
9.36	7.03
7.46	6.58
10.15	8.45
7.95	4.65
7.65	6.11
8.28	4.97
8.61	5.79
8.65	8.18
7.85	5.52
7.60	6.18
7.41	6.30
7.08	6.73
6.97	4.53
8.04	6.60
8.09	6.65
9.05	6.48
9.17	6.49
7.91	8.63
7.86	6.19
6.96	6.78
6.16	8.48

Table S2: *Concentration of karmitoxin per cell*

Comparison of karmitoxin concentration in picograms per cell in cultures grown in f/2 media and fed *Rhodomonas salina* (left column) and cultures grown in ammonium substituted f/2 media (right column).

References for Supporting Information

Cai, Pengjie et al. 2016. "Two New Karlotoxins Found in Karlodinium Veneficum (Strain GM2) from the East China Sea." *Harmful Algae* 58:66–73.

Doi, Yukiko et al. 1997. "Luteophanol A, a New Polyhydroxyl Compound from Symbiotic Marine Dinoflagellate Amphidinium Sp." *The Journal of Organic Chemistry* 62(12):3820–23.

Echigoya, Reiko, Lesley Rhodes, Yasukatsu Oshima, and Masayuki Satake. 2005. "The Structures of Five New Antifungal and Hemolytic Amphidinol Analogs from Amphidinium Carterae Collected in New Zealand." *Harmful Algae* 4(2):383–89.

Hanif, Novriyandi, Osamu Ohno, Makoto Kitamura, Kaoru Yamada, and Daisuke Uemura. 2010. "Symbiopolyol, a VCAM-1 Inhibitor from a Symbiotic Dinoflagellate of the Jellyfish Mastigias Papua." *Journal of natural products* 73(7):1318–22.

Houdai, Toshihiro et al. 2001. "Acetate Labeling Patterns of Dinoflagellate Polyketides, Amphidinols 2, 3 and 4." *Tetrahedron* 57(26):5551–55.

Huang, Shin-Jong, Chih-Ming Kuo, Ying-Chih Lin, Yi-Min Chen, and Chung-Kuang Lu. 2009. "Carteraol E, a Potent Polyhydroxyl Ichthyotoxin from the Dinoflagellate Amphidinium Carterae." *Tetrahedron Letters* 50(21):2512–15.

Huang, Xiao Chun, Di Zhao, Yue Wei Guo, Hou Ming Wu, Li Ping Lin, et al. 2004. "Lingshuiol, a Novel Polyhydroxyl Compound with Strongly Cytotoxic Activity from the Marine Dinoflagellate Amphidinium Sp." *Bioorganic and Medicinal Chemistry Letters* 14(12):3117–20.

Huang, Xiao Chun, Di Zhao, Yue Wei Guo, Hou Ming Wu, Enrico Trivellone, et al. 2004. "Lingshuiols A and B, Two New Polyhydroxy Compounds from the Chinese Marine Dinoflagellate Amphidinium Sp." *Tetrahedron Letters* 45(28):5501–4.

Hwang, Buyng Su et al. 2013. "Ostreol A: A New Cytotoxic Compound Isolated from the Epiphytic Dinoflagellate Ostreopsis Cf. Ovata from the Coastal Waters of Jeju Island, Korea." *Bioorganic & Medicinal Chemistry Letters* 23(10):3023–27.

Inuzuka, Toshiyasu, Kaoru Yamada, and Daisuke Uemura. 2014. "Amdigenols E and G, Long Carbon-Chain Polyol Compounds, Isolated from the Marine Dinoflagellate Amphidinium Sp." *Tetrahedron Letters* 55(46):6319–23.

Inuzuka, Toshiyasu, Yutaka Yamamoto, Kaoru Yamada, and Daisuke Uemura. 2012. “Amdigenol A, a Long Carbon-Backbone Polyol Compound, Produced by the Marine Dinoflagellate Amphidinium Sp.” *Tetrahedron Letters* 53(2):239–42.

Kubota, Takaaki et al. 1998. “Luteophanols B and C, New Polyhydroxyl Compounds from Marine Dinoflagellate Amphidinium Sp.” *Tetrahedron* 54(48):14455–64.

Kubota, Takaaki, Ayako Takahashi, Masashi Tsuda, and Jun’ichi Kobayashi. 2005. “Luteophanol D, New Polyhydroxyl Metabolite from Marine Dinoflagellate Amphidinium Sp.” *Marine Drugs* 3(4):113–18.

Meng, Yanhui, Ryan M. Van Wagoner, Ian Misner, Carmelo Tomas, and Jeffrey L. C. Wright. 2010. “Structure and Biosynthesis of Amphidinol 17, a Hemolytic Compound from Amphidinium Carterae.” *Journal of natural products* 73(3):409–15.

Morsy, Nagy et al. 2005. “Isolation and Structure Elucidation of a New Amphidinol with a Truncated Polyhydroxyl Chain from Amphidinium Klebsii.” *Tetrahedron* 61(36):8606–10.

Morsy, Nagy et al. 2006. “Structures of New Amphidinols with Truncated Polyhydroxyl Chain and Their Membrane-Permeabilizing Activities.” *Bioorganic & Medicinal Chemistry* 14(19):6548–54.

Murata, Michio, Respati T. Swasono, Mitsunori Kanemoto, and Tohru Oishi. 2010. “Structural Reevaluations of Amphidinol 3, a Potent Antifungal Compound from Dinoflagellate.” *HETEROCYCLES* 82(2):1359.

Nuzzo, Genoveffa, Adele Cutignano, Angela Sardo, and Angelo Fontana. 2014. “Antifungal Amphidinol 18 and Its 7-Sulfate Derivative from the Marine Dinoflagellate Amphidinium Carterae.” *Journal of Natural Products* 77(6):1524–27.

Paul , , Gopal K., Matsumori , Nobuaki, Keiichi Konoki, Michio Murata, and Kazuo Tachibana. 1997. “Chemical Structures of Amphidinols 5 and 6 Isolated from Marine dinof1agellate Amphidinium Klebsii and Their Cholesterol-Dependent Membrane Disruption.” *Journal of Marine Biotechnology* (September 2014).

Paul, Gopal K., Nobuaki Matsumori, Michio Murata, and Kazuo Tachibana. 1995. “Isolation and Chemical Structure of Amphidinol 2, a Potent Hemolytic Compound from Marine Dinoflagellate Amphidinium Klebsii.” *Tetrahedron Letters* 36(35):6279–82.

Peng, Jiangnan, Allen R. Place, Wesley Yoshida, Clemens Anklin, and Mark T. Hamann. 2010. “Structure and Absolute Configuration of Karlotoxin-2, an Ichthyotoxin from

the Marine Dinoflagellate Karlodinium Veneficum." *Journal of the American Chemical Society* 132(10):3277–79.

Rasmussen, Silas Anselm et al. 2017. "Karmitoxin: An Amine-Containing Polyhydroxy-Polyene Toxin from the Marine Dinoflagellate Karlodinium Armiger." *Journal of Natural Products* acs.jnatprod.6b00860.

Satake, Masayuki, Michio Murata, Takeshi Yasumoto, Tsuyoshi Fujita, and Hideo Naoki. 1991. "Amphidinol, a Polyhydroxypolyene Antifungal Agent with an Unprecedented Structure, from a Marine Dinoflagellate, Amphidinium Klebsii." *Journal of the American Chemical Society* 113(26):9859–61.

Sugahara, Kohtaro, Yoshiaki Kitamura, Michio Murata, Masayuki Satake, and Kazuo Tachibana. 2011. "Prorocentrol, a Polyoxy Linear Carbon Chain Compound Isolated from the Toxic Dinoflagellate Prorocentrum Hoffmannianum." *Journal of Organic Chemistry* 76(9):3131–38.

Van Wagoner, Ryan M. et al. 2008. "Isolation and Characterization of Karlotoxin 1, a New Amphipathic Toxin from Karlodinium Veneficum." *Tetrahedron Letters* 49(45):6457–61.

Van Wagoner, Ryan M. et al. 2010. "Structure and Relative Potency of Several Karlotoxins from Karlodinium Veneficum." *Journal of Natural Products* 73(8):1360–65.

Washida, Kazuto, Tomoyuki Koyama, Kaoru Yamada, Masaki Kita, and Daisuke Uemura. 2006. "Karatungiols a and B, Two Novel Antimicrobial Polyol Compounds, from the Symbiotic Marine Dinoflagellate Amphidinium Sp." *Tetrahedron Letters* 47(15):2521–25.

Waters, Amanda L., Joonseok Oh, Allen R. Place, and Mark T. Hamann. 2015. "Stereochemical Studies of the Karlotoxin Class Using NMR Spectroscopy and DP4 Chemical-Shift Analysis: Insights into Their Mechanism of Action." *Angewandte Chemie International Edition* 54(52):15705–10.
